# Vaccinia Virus G8R Protein: A Structural Ortholog of Proliferating Cell Nuclear Antigen (PCNA)

**DOI:** 10.1371/journal.pone.0005479

**Published:** 2009-05-07

**Authors:** Melissa Da Silva, Chris Upton

**Affiliations:** Department of Biochemistry and Microbiology, University of Victoria, Victoria, British Columbia, Canada; University of Hong Kong, Hong Kong

## Abstract

**Background:**

Eukaryotic DNA replication involves the synthesis of both a DNA leading and lagging strand, the latter requiring several additional proteins including flap endonuclease (FEN-1) and proliferating cell nuclear antigen (PCNA) in order to remove RNA primers used in the synthesis of Okazaki fragments. Poxviruses are complex viruses (dsDNA genomes) that infect eukaryotes, but surprisingly little is known about the process of DNA replication. Given our previous results that the vaccinia virus (VACV) G5R protein may be structurally similar to a FEN-1-like protein and a recent finding that poxviruses encode a primase function, we undertook a series of *in silico* analyses to identify whether VACV also encodes a PCNA-like protein.

**Results:**

An InterProScan of all VACV proteins using the JIPS software package was used to identify any PCNA-like proteins. The VACV G8R protein was identified as the only vaccinia protein that contained a PCNA-like sliding clamp motif. The VACV G8R protein plays a role in poxvirus late transcription and is known to interact with several other poxvirus proteins including itself. The secondary and tertiary structure of the VACV G8R protein was predicted and compared to the secondary and tertiary structure of both human and yeast PCNA proteins, and a high degree of similarity between all three proteins was noted.

**Conclusions:**

The structure of the VACV G8R protein is predicted to closely resemble the eukaryotic PCNA protein; it possesses several other features including a conserved ubiquitylation and SUMOylation site that suggest that, like its counterpart in T4 bacteriophage (gp45), it may function as a sliding clamp ushering transcription factors to RNA polymerase during late transcription.

## Introduction

Poxviruses comprise a family of large, double-stranded DNA viruses that replicate in the cytoplasm of the host cell; the viruses infect a wide range of hosts including birds, mammals and insects [1]. The replication of the poxvirus genome begins between 1 and 2 hours post-infection and ends after the formation of approximately 10,000 copies of the genome; up to 5,000 may be packaged into new virions [1]. Although several viral proteins have been assigned roles in the process of viral genome replication, generally it is still poorly understood. Whether the virus replicates its genome via a mechanism with leading and lagging strand DNA synthesis similar to eukaryotic DNA replication [2], or via a virus-specific mechanism, remains to be resolved. Sedimentation studies performed in 1978 found small viral DNA fragments covalently linked to RNA primers and, most recently, a DNA primase encoded by vaccinia virus (VACV) was identified and found to be capable of synthesizing RNA primers [3], [4]. Such studies support the hypothesis of a eukaryotic-like lagging strand DNA synthesis mechanism. Related to the second of these studies is our identification of a flap endonuclease-like protein (VACV strain Copenhagen G5R), which could function to remove RNA primers from Okazaki fragments during lagging strand DNA synthesis [5].

In eukaryotic DNA replication, synthesis of the lagging strand and the removal of RNA primers from Okazaki fragments require several proteins, including flap-endonuclease (FEN-1) and proliferating cell nuclear antigen (PCNA) [2]. The FEN-1 protein associates with the PCNA protein, which acts as a sliding clamp that moves along the DNA bringing the FEN-1 protein to the RNA primers that must be removed [6]. Coupling of FEN-1 to PCNA increases its activity 10 to 50 fold [6], [7]. The PCNA protein also interacts with a variety of other proteins (clients), which can be grouped into 3 categories: DNA replication, DNA repair and cell cycle regulation [8]–[10]. DNA replication proteins include DNA polymerase delta, FEN-1, and the clamp loading complex replication factor C (RFC) that functions to assemble the three subunits of PCNA around the DNA duplex [9]. DNA repair client proteins include FEN-1, Xeroderma pigmentosa G (XPG) protein and uracil DNA glycosylase [9]. Interestingly, VACV also encodes a uracil DNA glycosylase that functions as a processivity factor and interacts with the VACV A20R protein during genome replication [11], [12]. One example of a PCNA client involved in cell cycle regulation is the p21 protein, also known as WAF1 and Cip1, which interacts and inhibits cyclin dependent kinases [9], [13].

In this study, we investigated the vaccinia virus protein G8R as a possible poxviral analogue of PCNA. Secondary and tertiary structure modelling programs yielded structures that showed remarkable similarity between G8R and the human and yeast PCNA proteins. We conclude that there is good evidence that G8R adopts a sliding clamp structure and that it could function in a similar manner to PCNA.

## Results and Discussion

Since eukaryotic FEN-1 requires PCNA to function efficiently [6], [7], we hypothesized that VACV and other poxviruses might also encode a PCNA-like protein that would interact with the viral FEN-1-like protein. We began by searching all VACV proteins for any potential sliding clamp motifs using the InterProScan program [14], [15] via the JIPS interface [16]. The results of more than 250 searches indicated that only the VACV G8R protein contained a sliding clamp motif (SSF55979, Superfamily DB). Upon further inspection, we discovered that G8R matched the PCNA-like class of sliding clamps.

The VACV G8R gene encodes a 260 amino acid protein that is encoded in all chordopoxvirus genomes, is expressed at intermediate stages of infection, and has been shown to play a role in poxvirus late transcription [17]–[19]. The InterProScan search routine placed the G8R protein in two different functional categories: 1) *viral trans-activator protein* (InterProScan ID: IPR005022; derived from the above work) and 2) *DNA clamp*, which is part of the DNA clamp superfamily in the SCOP (structural classification of proteins) database (SCOP ID: 55979). This superfamily is itself subdivided into two families: the DNA polymerase III beta subunit family, including multiple domains from the *E. coli* DNA polymerase III beta subunit, and the DNA polymerase processivity factor family that contains sliding clamp proteins from several organisms, including herpes virus, human, yeast and 3 archaeal species.

A second set of independent evidence, comparing protein profiles, was used to confirm the similarity between VACV G8R and the sliding clamp proteins and to determine which category of sliding clamp the viral protein was most similar to. A multiple alignment of 16 diverse G8R protein orthologs, created using T-Coffee [20] with manual editing, was used as input for the HHsearch tool [21], [22]. Where InterProScan combines several different protein domain prediction tools to identify functional domains in the query sequence, the HHsearch tool creates a Hidden Markov Model (HMM) profile of the query sequence or alignment (first taking into consideration a prediction of the protein's secondary structure) and then compares this profile to the HMM profiles of all protein sequences in the PDB, SCOP and CATH databases [23]–[27]. The HHsearch tool reported the yeast PCNA protein as the best match, with a probability of 95.5%. With our hypothesis of a VACV sliding clamp protein now supported by two separate lines of bioinformatics evidence, we proceeded to analyze predicted secondary and tertiary structures for the G8R protein.

### Secondary and tertiary structure conservation

**Figure 1:**
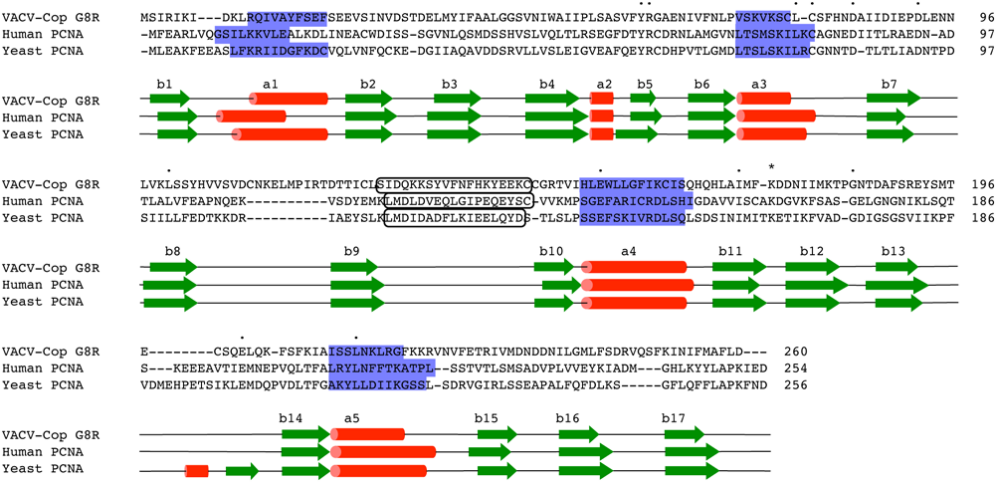
Comparative alignment between VACV G8R, human and yeast PCNA. Alignment created using T-Coffee alignment algorithm, default parameters, with minor adjustments made by hand [20]. Green arrows represent beta-strands, and red cylinders represent alpha-helices. Black rounded box shows the interdomain connecting loop, blue shaded boxes represent the helices that contribute to DNA binding and dots represent identical residues between the 3 protein sequences. Residue marked with an asterisk is the conserved ubiquitylation/SUMOylation site. Secondary structure was derived directly from the crystal structures of the human and yeast PCNA proteins and the structural model of the VACV G8R protein.

The T-Coffee alignment of the VACV G8R, human and yeast PCNA proteins (Figure 1) shows 35% amino acid identity between human and yeast PCNA and only 9% and 12% amino acid identity between the VACV G8R protein and the human and yeast proteins, respectively. Though this low sequence identity between the VACV G8R protein and these eukaryotic proteins is below what is often considered a cut-off for predicting functional similarity (20%), it is important to note that the amino acids conserved in G8R are a subset of the residues conserved between the human and yeast proteins. Furthermore, the same residues were found to remain conserved when additional diverse eukaryotic PCNA proteins were included in the multiple sequence alignment (data not shown). Recently, it was found that human PCNA can be modified at lysine 164 with either a ubiquitin or SUMO moiety [10] and this residue is also conserved in VACV G8R (lysine 174, Figure 1 marked with an asterisk).

The predicted secondary structure of the VACV G8R protein also showed remarkable similarity to the experimentally determined secondary structures of the human and yeast PCNA proteins (Figure 1). Studies of crystal structures for human and yeast PCNA have shown that these proteins form trimers with pseudo six-fold symmetry; each subunit has two key domains separated by the interdomain connecting loop [28]. For all three proteins (VACV G8R, human and yeast PCNA), the first domain has the secondary structure of: SHSSSSSHSSS (where S is a beta-strand and H is an alpha-helix). Although all three of the second domains are structurally very similar to the first domain, the VACV G8R and human PCNA proteins are missing one beta-strand and have the secondary structure: SHSSSSHSSS. The second domain in the yeast PCNA protein has a secondary structure identical to the first domain. The interdomain connecting loop (see box in Figure 1) consists of 18 amino acids for the VACV G8R and human PCNA proteins, and 17 amino acids for yeast PCNA.

**Figure 2:**
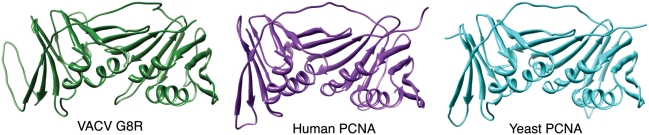
Structures of the VACV G8R (predicted), human and yeast PCNA (experimentally determined) proteins.

Given that the predicted secondary structure for VACV G8R also supported our hypothesis, we explored the possibility that this viral protein might adopt a tertiary structure similar to the experimentally determined PCNA structures. The Robetta protein structure prediction server [29], which utilizes comparative as well as *ab initio* approaches, was used to create a structural model of the complete G8R protein using the yeast PCNA crystal structure (PDB:1PLQ) as a template. The results of this modelling indicate that the VACV G8R protein can adopt a tertiary structure that is very similar to the crystallographic structures of both human and yeast PCNA proteins (Figure 2). The root-mean-square deviation (RMSD) values for the superposition of VACV G8R over the human and yeast PCNA crystal structures are 1.45Å over 228 alpha-carbon pairs and 0.74Å over 226 alpha-carbon pairs respectively. In comparison, the RMSD for the superposition of the human and yeast crystal structures is 1.24Å over 249 alpha-carbon pairs. These values show that despite the low sequence identity between the G8R and human and yeast proteins, the predicted tertiary structure of the G8R protein is as similar to the human PCNA structure as is the yeast PCNA structure (since the VACV G8R model was produced using the yeast crystal structure as a template, it is not surprising that there is a lower RMSD value between these two tertiary structures than between G8R and the human PCNA structure). This conclusion is also supported by the FUGUE sequence-structure comparison tool, which also identified PCNA as a distant homolog of G8R with 95% confidence (Z-score = 5.91) [30].

**Figure 3:**
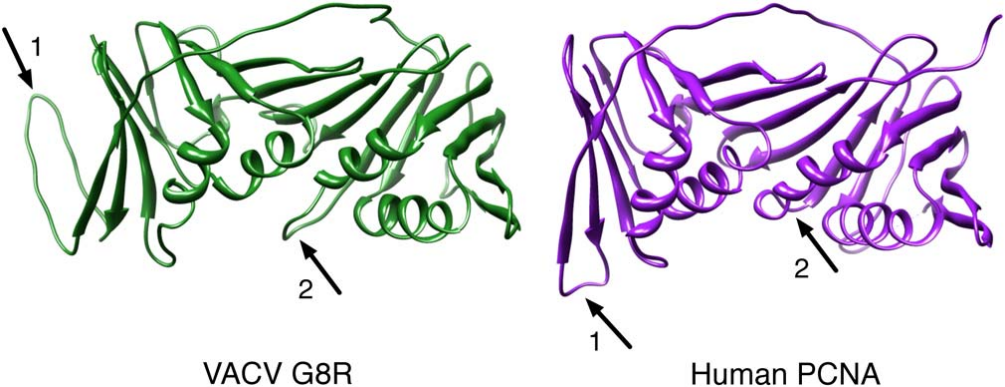
Two differences between the VACV G8R and human PCNA structures. Arrows labelled 1 mark the loop region between beta-strands 8 and 9; arrows labelled 2 mark the loop region between alpha-helix 5 and beta-strand 15.

The functional role of the eukaryotic PCNA protein is to act as a linker between DNA and a number of diverse client proteins; thus, PCNA does not have an obvious structural “active site” that is usually associated with proteins having enzymatic function. However, two interaction domains are present in PCNA, the DNA and client-binding domains, and these were further characterized in the following manner. Though the PCNA and predicted VACV G8R structures are extremely similar, there are two relatively subtle differences that distinguish them. The first is the loop region located between beta-strands 7 and 8 (Figure 3, arrow 1). In the VACV G8R structural model, this loop is 16 amino acids long and extends out towards the back of the protein whereas in human PCNA this loop is considerably shorter (only 6 residues) and extends downwards (Figure 3, arrow 1). Given that loops are the most difficult regions to predict in a structural model, speculation on the significance of this apparent difference is not feasible. However, as the protein sequence in this region is conserved in all chordopoxviruses with only minor amino acid differences (seen primarily in the avipoxviruses and molluscum contagiosum virus), it may play a significant functional role. The second difference is in the loop region modelled between alpha-helix 5 and beta-strand 15; it is 9 amino acids long in the VACV G8R protein and is only a 2 amino acid loop in human PCNA (Figure 3, arrow 2). Assuming correct modelling, the loop is seen to extend into the DNA binding region of the VACV G8R protein and may therefore play a role in stabilizing its interaction with DNA.

**Figure 4:**
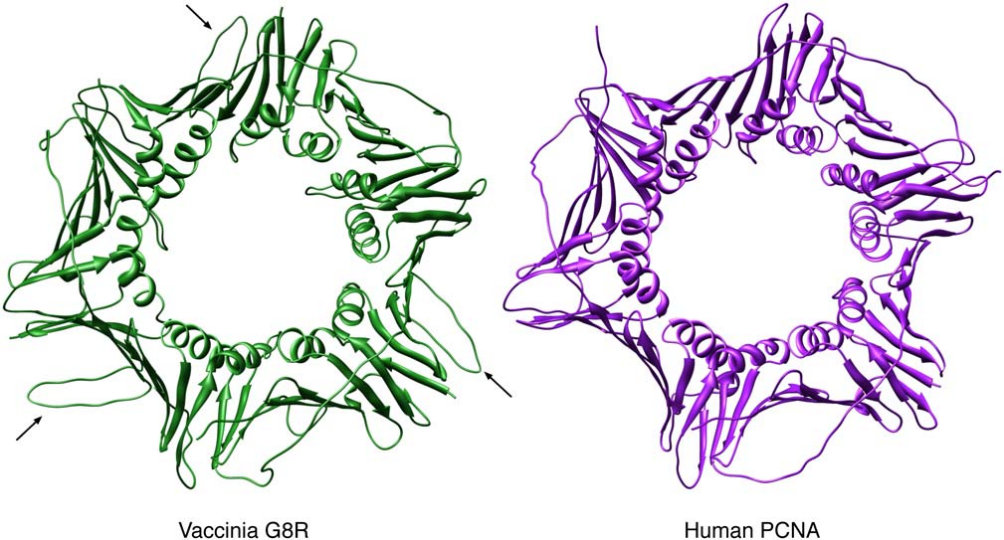
Complete trimer of the VACV G8R and human PCNA protein donut structures. Arrows mark the loop region between beta-strands 8 and 9 of the VACV G8R protein.

**Figure 5:**
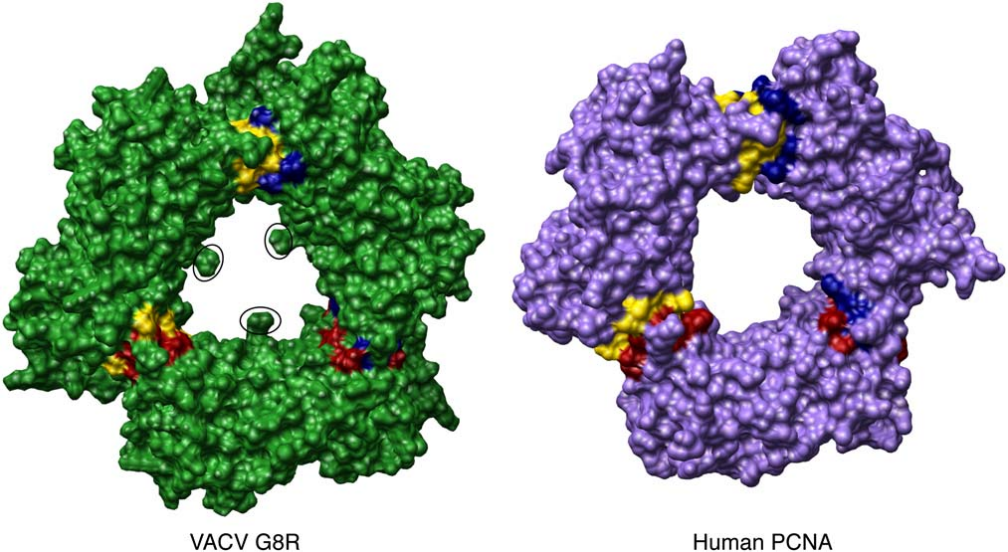
Surface diagrams of the VACV G8R and human PCNA trimers. Residues found to contribute to trimer stabilization and belonging to chain A of both structures are coloured in blue, chain B in yellow and chain C in red. Circles indicate the three residues on each VACV G8R monomer that protrude into the “donut hole” of the molecule.

The human PCNA protein functions as a homotrimer, essentially forming a donut-shaped clamp around the DNA [31]. We found that arranging three copies of the VACV G8R structural model using the human PCNA crystal structure as a template generated a structure similar to the human PCNA trimer (Figures 4 and 5). Of particular interest is the fact that the extended loop region seen between beta-strands 8 and 9 on the VACV G8R protein does not appear to hinder trimer formation (Figure 4). At first viewing, it appears that three residues (V223, N224, V225) on each subunit protrude into the “donut-hole” (Figure 5), but since these residues are in a loop region on the G8R subunit and, as such, cannot be modelled reliably, we believe this does not nullify our hypothesis. There is also biochemical evidence to support the formation of a PCNA-like structure; a yeast two-hybrid assay found that the VACV G8R protein is capable of forming strong interactions with itself [18]. Using the InterProSurf protein interaction prediction server, we also investigated which residues on each subunit might contribute to trimer stabilization [32] (Table 1, Figure 5). InterProSurf predicted 18 residues in the VACV G8R subunit and 21 residues in the human PCNA subunit that could potentially contribute to the trimer stability (Table 1, Figure 5). The validity of such analyses is supported by the fact that the results obtained for human PCNA are residues previously known to play a role in trimer stabilization [33].

**Table 1:** Residues and positions of the amino acids in both VACV G8R and human PCNA that are predicted to play a role in trimer stabilization.

VACV G8R	Human PCNA
Residue and position	Residue and position
K73	S74
S76	K77
S80	I78
H82	C81
T119	E109
T121	K110
I123	D113
C124	Y114
K160	E115
S163	K117
Q164	D150
T185	H153
D186	I154
A187	L175
F188	G176
S189	N177
R190	G178
S193	N179
	I180
	L182
	S183

### Interaction domain comparison

The PCNA protein contains two interaction domains (not to be confused with the structural domains discussed above); one binds client proteins and the other binds DNA. The client binding domain consists of a long protein loop (the interdomain connecting loop) that connects the two structural domains (Figures 1 and 2) and interacts with client proteins that contain a PCNA interacting protein (PIP) motif (Qxx[M/L/I]xxF[Y/F], where x is any amino acid). In the human FEN-1 client protein, this motif is located at the C-terminus, although in some client proteins, the PIP motif may be located at the N-terminus or even near the middle of the protein and exposed on its surface [34]. Although the similarity of VACV G5R protein to FEN-1 initiated this search for a poxvirus PCNA-like protein, there is no direct evidence to support G5R being a client for the poxvirus G8R PCNA-like protein. To date, G5R has not been shown to interact with G8R, whereas A1L, A2L and H5R do interact with G8R [18]. Inspection of the surface of the VACV G5R structural model reveals several exposed phenylalanine residues that are reminiscent of a PIP motif, but there is no PIP motif in G5R. However, since the client binding domains of the VACV G8R, human, and yeast PCNA proteins differ significantly in sequence, it is perhaps not surprising that the G5R protein does not contain an exact PIP motif, and wet-lab experiments should be performed to determine if there are conditions in which G5R and G8R interact. As noted above, VACV G8R, like PCNA, interacts with several other proteins (A1L, A2L and H5R) [18], this further supports our hypothesis that VACV G8R shares structure and some functionality with PCNA.

**Figure 6:**
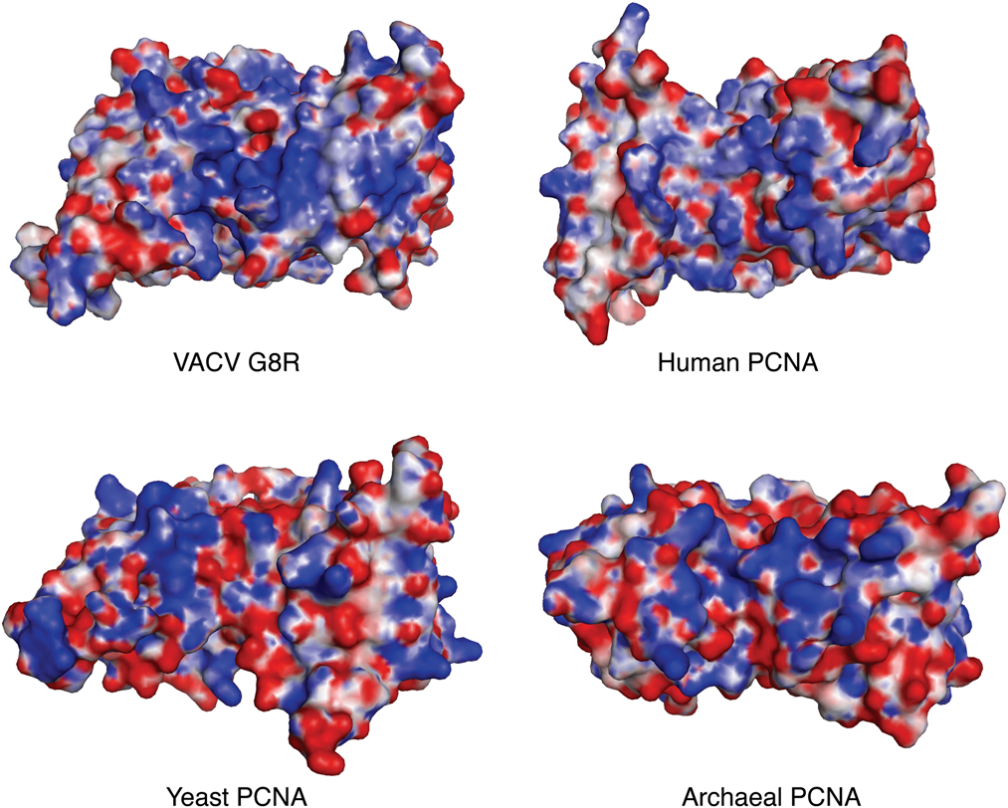
Electrostatic surface diagrams of the VACV G8R protein and 3 other PCNA proteins. Surface diagrams are coloured by electrostatic properties as calculated by APBS using all default values with positively charged regions coloured in blue and negatively charged regions in red.

The second functional domain in PCNA is the DNA binding domain, consisting of the 4 alpha helices that cluster towards the centre of the trimer and surround the hole into which the DNA fits (Figure 1 blue shaded boxes, and Figure 4). As is common in DNA binding proteins, these alpha helices contain positively charged amino acids that interact electrostatically with the negatively charged DNA [35]. The number of lysine and arginine residues contained in these four helices ranges from 7 and 9 over a typical set of eukaryotic and archaeal PCNA proteins; the VACV G8R protein contains 6 positively charged residues. To get a better idea of the locations of these and other residues that contribute to overall surface electrostatic charge, the electrostatic potential of the human, yeast, archaeal and G8R proteins was calculated and mapped onto surface representations of each protein (Figure 6). These results reveal that the electrostatic potential of the helical region of the VACV G8R protein is similar to that seen for the other 3 PCNA proteins. The positively charged residues tend to cluster near the centre of the helical region for the G8R protein, as is also observed in the archaeal PCNA protein (Figure 6). The human and yeast proteins both have some positively charged residues near the centre of the helical region, but overall demonstrate a less localized charge pattern than that seen in the archaeal and G8R proteins. Given that the arrangement of positive charge observed in the G8R protein is consistent with that seen with the other three proteins, it is quite possible that the G8R protein is also capable of binding to DNA although further biochemical experiments are required to prove this hypothesis. Given the role that the G8R protein plays in poxvirus late transcription, it is perhaps not surprising that it may be able to bind DNA, but whether it plays a role solely in transcription or shows properties similar to those of PCNA remains to be determined.

### Conclusions

The data presented here demonstrate that the predicted secondary and tertiary structures of the VACV G8R protein are very similar to those of the highly conserved eukaryotic PCNA protein, which functions as a DNA sliding clamp. The similarity in predicted secondary structure and the ability to fold the VACV G8R polypeptide onto the experimentally determined PCNA tertiary structure is strong evidence that these proteins adopt similar 3D structures. There is also evidence to support the hypothesis that the VACV G8R protein may adopt a quaternary structure similar to that of the PCNA homotrimer. First, laboratory data shows that the VACV G8R protein interacts with itself [18]. Second, 10 of the 18 amino acids identified by InterProSurf software [32] as being potentially involved in the stability of a modelled VACV G8R trimer are absolutely conserved among all of the poxvirus G8R orthologs. Third, the distribution of the positively charged residues in the helical region of the G8R protein is very similar to the DNA binding domains of the PCNA proteins. Fourth, the VACV G8R protein also interacts with A1L, A2L and H5R [18] providing the possibility that these may represent vaccinia analogs of PCNA client proteins. Finally, all of these structural characteristics exist with very minimal amino acid sequence conservation, enhancing the significance of the structural findings.

Although the data presented here indicates that the VACV G8 protein is structurally similar to PCNA proteins, one needs to be aware that if these are true homologs then they have been evolutionarily separated for a very long time. This may translate to loss and/or gain of function with respect to contemporary PCNA proteins and it is likely that the role of VACV G8R is not a simple mirror of PCNA function in eukaryotes. For example, the VACV uracil DNA glycosylase-like (UNG) protein [11], [36] is distinguished from the UNGs of other organisms in that it has evolved into an essential gene, with a role in processivity of DNA replication. Interestingly, there are no orthologs of VACV UNG or G8R in the Entomopoxviruses (Entomopoxvirus UNGs are more similar to bacterial and herpesvirus UNGs [37], [38]), thus these two essential proteins, involved in DNA/RNA, belong to a set of genes that are in all Chordopoxviruses and absent from the Entomopoxviruses.

The modeling of the G8R structure is only one step in determining the function of this protein, other observations add much to the goal. The G8R protein does not appear to be required for synthesis of new viral DNA since 1) it is encoded by an intermediate gene [39] and therefore expressed after initiation of DNA replication, and 2) experiments using an inducible conditional-lethal mutant VACV [39] to repress G8R synthesis also demonstrated that new viral DNA accumulates although concatenated genomes are not resolved [39]. However, an interaction partner of G8R, VACV H5R [40], [41], has recently been identified as playing an essential role in several steps of poxvirus DNA replication (Dr Rich Condit, personal communication). H5R is also known as a late transcription stimulatory factor [40] and interacts with the elongation factor G2R [42] as well as replication proteins A20R and B1R [43]. Thus, H5R sits at a junction between poxvirus transcription and replication. Although DNA synthesis can take place in the absence of G8R, the interaction between G8R and H5R indicates that it is, in some way, linked to replication as suggested by its PCNA structure.

There is a well-characterized precedent for a sliding clamp protein to function in both replication and transcription. Protein gp45 of bacteriophage T4 acts in replication and as an activator of late transcription [44]–[46]. Despite very little sequence similarity to PCNA, gp45 is remarkably similar in structure, forming the donut-like sliding clamp by assembling a trimer of the two-domain subunits [47]. It is also curious that the consensus sequence for T4 late promoters is TATAAATA [46], whereas the poxvirus late promoters are TAAATG [48].

The evidence presented in this study demonstrates that the VACV genome encodes a PCNA-like protein and suggests that it may form a sliding clamp-like structure. The parallels with the T4 system are intriguing, and also may help direct new experiments to determine the mechanisms by which these poxvirus proteins function in replication and transcription. For example, the T4 gp45 protein uses a two-protein loader-complex and assembles at DNA structures such as nicks or gaps rather than on specific sequences [49]. Clearly, experimentally derived structures of these proteins and the determination of those amino acids involved in VACV G8R-binding partner interactions would be very useful in showing if the complex resembles PCNA-client or T4 gp45 complexes, or whether they are poxvirus-specific.

## Methods

The initial VACV genome-wide InterProScan [15] was performed using the JIPS program developed in our laboratory [16]. Results of the scan were manually parsed looking for the keywords “sliding clamp”. The one protein (VACV G8R) to fit this criteria was examined in further detail at the InterProScan website (http://www.ebi.ac.uk/Tools/InterProScan/).

A T-Coffee [20] alignment of 16 poxvirus G8R sequences was used as input in the HHsearch HMM comparison search tool [21] and the VACV G8R sequence was used as input in the Robetta protein structure prediction server [29], [50]–[52]. Robetta successfully created 5 potential structural models of the VACV G8R protein using the crystal structure of yeast PCNA (PDB:1SXJ) [53] as a template. Comparisons were subsequently made to the crystal structure of human PCNA (PDB:1UL1) [28]. Protein structures were visualized and superimposed using the Chimera visualization software [54]. Electrostatic surface images were created with the APBS plug-in for PyMOL (http://www.pymol.org/) with positive potential coloured in blue and negative potential in red. Predictions of which residues within the G8R and human PCNA protein structures contribute to trimer stabilization were performed using InterProSurf [32], using default parameters.

The comparative alignment between VACV G8R, human and yeast PCNA protein sequences was created using T-Coffee with manual manipulations to ensure that the secondary structure that was being mapped contained no gaps. The secondary structure displayed with the alignment was derived directly from the crystal structures of the human and yeast PCNA proteins and from the model of the VACV G8R protein.
